# miR-137 Inhibition of the Invasion, Metastasis, and Epithelial-Mesenchymal Transition of Nasopharyngeal Cancer by Regulating KDM1A

**DOI:** 10.1155/2021/6060762

**Published:** 2021-12-17

**Authors:** Han-qiang Lu, Run-kun Wang, Hui-rong Wang, Guang-quan Zhou, Yan-shu Zhang

**Affiliations:** ^1^Department of Otorhinolaryngology, Affiliated Hospital of Jiangsu University, Zhenjiang 212001, China; ^2^The Fourth Affiliated Hospital of Nantong University, YanCheng 224006, China; ^3^The First People's Hospital of Yancheng, YanCheng 224006, China

## Abstract

One of the most frequent malignancies in the head and neck is nasopharyngeal carcinoma (NPC). MicroRNAs, a kind of tiny noncoding RNA molecule, have been used as negative regulators in different types of cancer therapy in recent decades by downregulating their targets. Recent research suggests that microRNAs play an important role in cancer's epithelial-to-mesenchymal transition (EMT), supporting or inhibiting EMT development. The epithelial-to-mesenchymal transition (EMT) is linked to a variety of cancer-related activities, including growth, metastasis, and invasion. Previous research has linked EMT to cancer stem-like characteristics as well as treatment resistance. Moreover, since microRNAs (miRNAs) are important regulators of the EMT phenotype, certain miRNAs have an effect on cancer stemness and treatment resistance. As a result, both fundamental research and clinical therapy benefit from knowing the connection between EMT-associated miRNAs and cancer stemness/drug resistance. As a result, we looked at the different functions that EMT-associated miRNAs (miR-137) play in the stem-like characteristics of malignant cells in this article. Then we looked at how EMT-associated miRNAs interact with nasopharyngeal cancer's drug-resistant complex signaling pathways. Using qRT-PCR, we evaluated the performance of several micro RNAs with the proposed miR-137 for inhibiting invasion, metastasis, and the EMT process. In conclusion, our findings showed that miR-137 acted as a tumor suppressor gene in controlling NPC EMT and metastasis and that it may be a new therapeutic strategy and prognosis marker for the disease.

## 1. Introduction

Nasopharyngeal carcinoma (NPC) is a rare epithelial cancer with a specific geographic and ethnic distribution in Asian countries, lymphoepithelioma-like histologic characteristics, and an etiological link to EBV (Epstein–Barr virus) infection. For most areas of the globe, the ASR (age-standardized rate) for this uncommon form of neck and head cancer is 1 per 100,000 per year. The significant ethnic variations in NPC tumorigenesis suggest that genetic predisposition plays a significant role in this illness. HLA class I genes have continuously been linked to NPC risk in epidemiological research and current large-scale genome-wide association studies (GWAS). Because the HLA class I genes encrypt enzymes that recognize and current international antigens for the initiation of a host immune system against diseased or malignant molecules, it is thought that high-risk populations with many specific HLA haplotypes may have a harder time mounting an immune reaction against persistent EBV infection in the nasopharyngeal epithelium, in which the disease begins [1].

MicroRNAs, also known as miRNAs, are tiny noncoding RNA molecules with approximately 22 nucleotides that act as downregulation to prevent target genes from being expressed. MiRNAs with base-pairing capacity may attach to the 3′ untranslated region (UTR) of their gene expression, causing target mRNAs to be degraded or translation restriction. In particular, complete complementarity between miRNA and its targets is likely to result in mRNA destruction, while poor complementary binding suggests translational inhibition of the targets. One miRNA may interact with many mRNAs, and multiple miRNAs can work together to control target genes, making miRNAs the most important regulator in cells. MiRNAs in cancer may influence cell growth, motility, invasion, cell survival, antibiotic resistance, angiogenesis, tumor development, and even metastasis, among other things. MiRNAs have been discovered to have important roles both as oncogenic genes (oncomiRs) and tumor suppressor genes (TsmiRs) in the formation of EMT since a large number of research progressively began to uncover activities of miRNAs on the EMT process of cancer [2]. Brain activity and neural signaling impairments are also linked to miR-137. As a whole, the finding demonstrates that miR-137 is critical for proper neural development and neuronal activity and also for healthier brain activity and that interruption of this miRNA could influence neuro-developmental procedures and synaptic activity, potentially contributing to schizophrenia pathophysiology. In gestational diabetes mellitus (GDM), miR-137 reduces the stability and movement of trophoblast cells by suppressing FNDC5 that might contribute to the pathophysiology of placenta cells and the incidence of unfavorable pregnancy complications.

The epithelial-mesenchymal transition (EMT) is a transformation of epithelial cells into mesenchymal stem cells with increased invasive and migratory abilities. Depending on the distinct biological contexts as well as operational implications, EMT has been divided into three subgroups. The “type three” EMT, also known as oncogenic EMT, is connected to the development of cancer. Epithelial-like cells are immobile and lack adherent junctions, while mesenchymal-like cells, which lack cell polarization, may facilitate the migration and invasion of primary tumor cells from one location to another. E-cadherin is an epithelial marker that regulates cell-cell contact. As mesenchymal markers, N-cadherin and vimentin have been discovered. When cancer cells transition from E- to N-cadherin, they migrate, invade, and metastasize more aggressively. Through promoting an E-cadherin to N-cadherin transition, slug transcription factors and snail may encourage epithelial cells to migrate and convert into mesenchymal cells [3].

When it comes to cancer, miRs may be either tumor-promoting or tumor-suppressing. During cancer progression, onco-suppressor miRs that block cancer cell invasion are downregulated. Downregulation of factors involved in cancer cell migration may be aided by increasing the expression of such miRs. Under hypoxic circumstances, this miR blocks the expression of PD-L1 (programmed death-ligand 1), which suppresses cancer. Several investigations have demonstrated that miRs are effective upstream mediators that address a variety of biological pathways. Increasing the production of tumor suppressor miRs may be a beneficial approach, and considerable research is presently being conducted to explore this possibility. On the other hand, oncogenic miRs may increase cancer cell aggressiveness and proliferation and are linked to a bad prognosis. In cancer treatment, their downregulation is of interest. As a result, targeting miRs may be a viable option for cancer treatment [4].

In this paper, we inhibit the invasion, metastasis, and epithelial-mesenchymal transition of nasopharyngeal cancer by micro RNA-137. The further part of the paper is organized as follows. Part II provides the related literature works. Part III explains the flow of the proposed methodology. Part IV analyzes the performance of the proposed work. Finally, part V concludes the overall idea of the paper.

## 2. Related Works

TGF, which is thought to induce EMT in a variety of malignancies, has been shown to activate EMT and enhance the invasive and migratory abilities of NPC cells, according to Huang et al. [5]. TGF-induced EMT, invasion, and migration were all reversed when SMAD4, a key component of the canonical TGF pathway, was inhibited. Additional research showed that miRNA-34a, which was shown to be downregulated in NPC tissues and inhibited NPC cell metastasis in vivo, was indeed the target of SMAD4. TGF-induced EMT development, migration, and invasion were similarly slowed by miRNA-34a overexpression, which inhibited SMAD4. Restoration of SMAD4 transcription, on the other hand, reversed the inhibitory effects of miRNA-34a on carcinogenesis. All of these findings showed that miRNA-34a inhibited invasion, TGF-induced, and migration of NPC cells by directly targeting SMAD4, suggesting that miR-34a may be used as a targeted therapy for NPC.

Pretreatment 18F-FDG PET/CT metabolic characteristics in nonendemic EBV (Epstein–Barr virus) DNA-related nasopharyngeal cancer (NPC) patients were treated with curable IMRT (intensity-modulated radiation therapy) with or without chemotherapy that were investigated by Alessi et al. [6].

Zhao et al. [7] discovered that NPC tissues and cell lines have lower miR-92b production. The clinical data revealed that low miR-92b production was clearly linked to a poor prognosis. We also verified that miR-92b was a new independent prognostic signal for determining NPC patients' 5-year mortality. Overexpression of MiR-92b reduced cell motility, invasion, and EMT progression, while miR-92b knockdown reversed the effects. Furthermore, miR-92b may regulate Smad3 by attaching directly to its 3′-UTR. MiR-92b was shown to act as a tumor suppressor gene in controlling NPC EMT and metastasis by activating Smad3, suggesting that it may be a novel therapeutic focus and prognosis marker for the disease.

Nasopharyngeal carcinoma is one of the most frequent malignant tumors in the head and neck, according to Wang et al. [8]. The discovery of potential miRNA biomarkers may greatly aid in the early diagnosis of nasopharyngeal cancer. Two microarray profiling data sets from the Gene Expression Omnibus (GEO) database were used to acquire miRNA expression profiles and clinical information. Univariate and multivariate Cox survival analysis, median absolute shrinking, and choice operators Cox regression analysis were used to create the miRNA signature model. This model has a higher AUC than previously reported models. According to the findings, a three-miRNA signature may be a new predictive biomarker for nasopharyngeal cancer.

Guo et al. [9] investigated the role of microRNA153 (miR153) in the viability of nasopharyngeal cancer (NPC) cells and the molecular basis that underpins it. When compared to paracarcinoma material, the production of miR153 in NPC patients was significantly lower. In 1309B cells, overexpression of miR153 reduced cell viability, caused apoptosis, raised caspase-3 and caspase-9 activities, and elevated the Bcl2 associated X protein/Bcl2 protein expression ratio. Transforming growth factor 2 (TGF2) and Smad2 protein expression were similarly reduced in 139B cells when miR153 was upregulated. In 1309B cells, a TGF2 inhibitor increased the impact of miR153 overexpression on cell viability reduction, inducing apoptosis, caspase-3 and caspase-9 activities, and the Bax/Bcl2 expression levels ratio.

Chen et al. [10] investigated the function of miR-299 in nasopharyngeal carcinoma and its treatment significance. In this research, the NP460 normal nasopharyngeal cells and the HK1, CNE1, SUNE1, and CNE2 nasopharyngeal tumor cell lines were utilized. For the expression levels, qRT-PCR was utilized. The MTT test was performed to determine cell viability.

Lv et al. [11] used quantitative real-time PCR to examine the expression of miR-28-3p/-5p in human NPC tissues. MiR-28-3p/-5p mimics overexpressed and inhibitor-suppressed miR-28-3p/-5p. Cultured HONE-1 cells were used to investigate the functions of miR-28-3p/-5p in NPC formation. In contrast to neighboring normal tissues, miR-28-3p and miR-28-5p mRNA increased expressions were substantially lower in NPC tissues. MiR-28-5p overexpression inhibited NPC cell growth and caused cell cycle arrest and death, while miR-28-3p enhanced NPC cell motility and invasion. The miRNAs affected various signaling pathways: miR-28-5p influenced the PI3K/AKT signaling system by altering cyclin D1 expression. MiR-28-3p, on the other hand, suppressed Nm23-H1 and increased EMT.

The goal of Baxi et al. [12] was to see whether miRNAs might be used as prognostic indicators in NPC. This systematic review and meta-analysis research followed PRISMA standards. The search technique, which was restricted to articles from 2012 January to 2018 March, utilized permutations of several “search keywords.” Two reviewers conducted a thorough examination of the retrieved papers, which included multilevel filtering and confirmation by other authors. Meta-analysis was carried out utilizing recently reported research' hazard ratios (HR) and 95% confidence intervals (CI) of mortality.

Kong et al. [13] investigated the amount of miR-193a-3p in radio-sensitive CNE-2 and radio-resistant CNE-1 NPC cell lines and identified SRSF2 to be the target gene of miR-193a-3p based on a literature study. They used miR-193a-3p-mimic or antagomiR to investigate SRSF2 activity at the proteins and mRNA stages. Finally, signaling pathway analysis was conducted to determine the function of miR-193a-3p/SRSF2 in signaling pathways.

Yang et al. [14] used RT-PCR and methylation-specific PCR on nasopharyngeal cancer tissues to identify miR-335 production and methylation. In nasopharyngeal cancer tissues with promoter methylation, miR-335 expression was substantially lower than in those with promoters unmethylation. The methylation of the miR-335 gene promoter was found to be greater in 14 (46.7%) of the 30 nasopharyngeal carcinoma tissues. Similarly, individuals with cervical lymph node metastases showed a greater incidence of methylation in the miR-335 promoters (66.7% vs. 16.7%) than those who did not have cervical lymph node metastases.

The goal of Wen et al. [15] was to find diagnostically meaningful circulating miRNA profiles in NPC patients. A unique microarray was used to extract total RNA from whole blood samples collected from 120 cases with NPC, 30 cases with head-neck tumors (HNT), and 30 HSs (healthy subjects). The microarray-identified expression levels of four miRNAs were confirmed using quantitative real-time reverse transcription response. The discovered miRNAs may be used as new serological biomarkers strategies for NPC.

miRNA transcript study in blood flow for the discovery of new signatures, according to Zou et al. [16], may aid in the early diagnosis of nasopharyngeal cancer (NPC) patients. The Exiqon miRNA qPCR panel was used to identify potential miRNAs during the screening step. Serum samples from 208 patients diagnosed and 238 healthy individuals were divided into three periods for further verification of differentially expressed miRNAs using qRT-PCR, testing, and quality assessment. The miRNA patterns found in clinical specimens and serum-derived exosomes samples were investigated further (32 NPC vs. 32 NCs).

Yu et al. [17] investigated the method by which PinX1 controls epithelial-mesenchymal transition (EMT) and metastatic spread in NPC, as well as its biological and clinical relevance in disease development. In nasopharyngeal CD133+ cancer stem cells, they discovered that overexpression of PinX1 and P53 reduced cell growth, invasion, and migration, but that inhibiting miR-200b prevented these effects (CSCs).

ZNF154 was identified by Hu et al. [18], using a genomic sequence DNA methylation screening method, although its methylation state and functions in NPC have yet to be explored. Methylation-specific PCR and quantitative Sequenom MassARRAY were used to determine the methylation status of ZNF154 in NPC. Wound healing and transwell invasion tests were used to test the invasions and migratory abilities. An artificial metastasis test in vivo was used to determine the function of ZNF154 in NPC metastasis. Protein alterations were investigated by western blotting, which was accompanied by ZNF154 under. The relationship between ZNF154 prognosis and methylation in NPC was investigated using the Kaplan–Meier methodology.

The goal of Zhu et al. [19] was to examine the precise processes of NPC cell migration, invasion, and metastasis. The higher expressions of miR-184 and Notch2 were confirmed using qualitative reverse-transcription PCR (qRT-PCR) and western blot. In vitro cellular uptake and wound-healing experiments were then used to investigate NPC cell invasion and migration. The target genes of miR-184 were validated using microRNA (miRNA) gene transcription predictions databases and a dual-luciferase reporting experiment.

cDNA microarray data was utilized by Liu et al. [20] to evaluate APLNR levels of expression in NPC tissues. When comparing NPC tissues to noncancerous nasopharyngeal epithelial tissues, we discovered that APLNR transcription was lower in NPC tissues. Following that, a large-scale sample of 1,015 tissues was utilized to confirm the finding and investigate the link between APLNR transcription and NPC prognosis.

Huang et al. [21] discovered that miR-137 is reduced in hepatocellular carcinoma (HCC) cell lines and has an inhibition activity on HCC migration and invasion in vitro. In HCC cells, EZH2 was a direct downstream target gene of miR-137, which inhibited invasion and migration by inhibiting EZH2-STAT3 signaling. Moreover, EZH2 overexpression restored the inhibitory activity of miR-137 copies on HCC cell motility and invasion. Furthermore, through inhibiting EZH2-STAT3 signaling, miR-137 decreased HCC lungs metastasis in vivo.

## 3. Proposed Work

**Figure 1:**
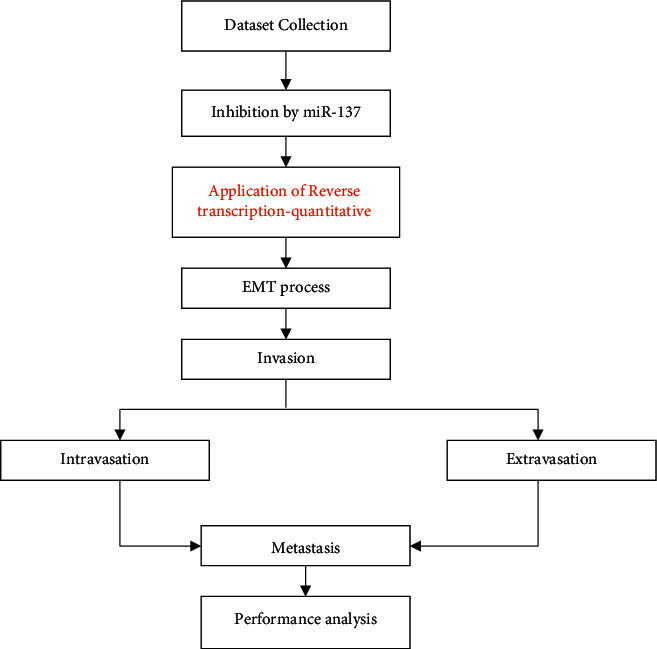
Flow of the proposed method.

One of the most frequent malignant tumors in the head and neck is nasopharyngeal carcinoma. The discovery of potential miRNA biomarkers may greatly aid in the early diagnosis of nasopharyngeal cancer. We attempted to investigate the biological role of miR-137 in NPC development in this research.  Figure 1 shows a schematic depiction of the suggested approach.

### 3.1. Data Set Description

The GEO (https://www.ncbi.nlm.nih.gov/geo) database was used to acquire miRNA expression profiles and clinical data for GSE32960 and GSE70970. Twelve 312 nondistant metastatic nasopharyngeal tumors and 18 noncancerous nasopharyngitis biopsy samples are included in the GSE32960 given data set. The average length of follow-up was 62.2 months (IQR 47.7–71.5). Every one of these samples was obtained between January 16, 2003, and February 25, 2006, at the Sun Yat-sen University Cancer Institute in Guangzhou, China. The US Joint Committee on Cancer Stage standards were used to classify the clinical staging (seventh version). A total of 246 cases with nasopharyngeal carcinoma from Princess Margaret Cancer Center are included in the GSE70970 data set (Toronto, Canada). The batch effect was eliminated using ComBat [8], and samples having a survival duration of less than one month were deleted.

### 3.2. Inhibition by miR-137

Although miR dysregulation has been linked to cancer in humans, the precise regulatory mechanism of miR137 in nasopharyngeal cancer is yet unclear. MiR137 was investigated in this research utilizing reverse transcription-quantitative polymerase chain reaction. In NPC, it may also decrease tumor development and increase paclitaxel and cisplatin sensitivities.

### 3.3. qRT-PCR Stands for Reverse Transcription-Quantitative PCR

Cells had their whole RNA harvested. Using the PrimeScript RT reagent kit, complementary DNA (cDNA) was generated from 500 ng total RNA. On the ABI PRISM® 7900 HT Rapid Real-Time PCR Technology, qPCR was conducted using the SYBR Premix ExTaq kit. qPCR was performed using the following thermocycling conditions: preincubation at 95°C for 30 seconds, followed by 45 cycles of 5 seconds at 95°C and 30 seconds at 60°C, with cooling at 40°C for 30 seconds in between. The primer combinations used are given as follows.

The 2Cq technique was used to determine relative fold expressions and variations. The internal audit for mRNA normalization was GAPDH shown in Table 1.

As the unspliced form of the ERR gene transcript, the amounts of ERR precursor mRNA (pre-ERR) were assessed using qRT-PCR. Exon 1 and the intron after it may be amplified using the primers employed. The forward to reverse primer sequencing for pre-ERR were 5′GCG ATG TCC TTT TGT GTC CT3′ and 5′CCT GAA CCC TGA CCA GTC C3′, respectively.

The TaqMan MicroRNA Reverse Transcription kit was used to produce cDNA to assess the expression levels of microRNAs (miRNAs/miRs). The following were the thermocycling conditions: 95°C for 3 minutes, then 40 cycles of 15 seconds at 95°C, and 30 seconds at 60°C. A forward primer (http://www.mirbase.org/search.shtml) was the precise sequencing of the matured miRNA. The forward primer for U6 has the following sequence: 5′-TGC GGG TGC TCG CTT CGC AGC-3′, whereas the reverse primer was provided by the abovementioned kit. The internal control for miRNA normalization was U6. The 2Cq technique was used to measure gene expression levels. All of the tests were carried out in threes.

### 3.4. Epithelial-Mesenchymal Transition (EMT)

After the dysregulation of epithelium characteristics, epithelial cells adopt mesenchymal phenotype and behaviors, known as epithelial-mesenchymal transition (EMT). EMT is induced by signals received by cells from their surroundings. Persistent epithelial cell-cell connections, apical-basal polarisation, and contacts with the basal cells characterize the epithelial condition of the tissues in which EMT is started. Genetic alterations and post-translational regulatory measures cause epithelial traits to be repressed and mesenchymal features to be acquired during EMT. The cells develop fibroblasts shape and cytoarchitecture and also greater migration ability. EMT is now commonly considered to occur naturally throughout early embryonic development to allow for a range of morphological processes and also later in growth and during adulthood wound repair. Furthermore, EMT has been linked to cancer etiology and tissue fibrosis. MET, or mesenchymal-epithelial transition, is a process that happens regularly during maturation. Because of this, cells that undergo EMT in vivo and retain both epithelial and mesenchymal characteristics are more able to persist in an intermediate state, rather than fully converting from one state to the other.

### 3.5. Invasion and Metastasis Inhibition

The spreading of cancerous cells to vital organs outside of the tumor's original location, as well as the production of additional tumors, is the single incident that triggers most cancer patients to die. N 50% of cancer patients had a clinically apparent metastatic illness at the time of diagnosis. A greater number of patients will have micrometastases that are undetectable by standard methods. As a result, metastasis is the most life-threatening occurrence in patients with cancer. The so-called metastatic cascading is made up of a series of consecutive processes that must occur in order for the tumor cell to properly spread. As a multiplexed illness, cancer is made more complicated by this mechanism. The relevance of alterations in cell-cell and cell-matrix adhesion during the metastatic process cannot be overstated [14].

Invasion, intravasation, and extravasation are the three primary mechanisms that make up the metastatic cascades. Malignant tumor cells detach from the initial tumor site due to a loss of cell-cell adhesion ability, and alterations in cell-matrix interaction enable the cells to infiltrate the adjacent stroma; this is the invasion mechanism. This includes the production of chemicals that destroy the basement membrane and extracellular matrix, as well as the expression/suppression of proteins that regulate movement and migration. Local dispersion for delivery of nutrition to and elimination of waste products from the target tissue would sufficient for tumors up to 2 mm diameter, but the tumor could fail to develop if angiogenesis was not initiated. The detached cells can then enter the circulatory system and spread to distant areas via a blood artery in the tumor's vicinity, a process known as intravasation. In the development of tumor angiogenesis, the interaction between the tumor cell and the adjacent stroma is crucial. When a tumor cell reaches a potential intravasation site, it engages in metabolic connections with endothelium, establishes adherence to the endothelium to build stronger attachments, and thereby breaches the endothelium and foundation membranes, a process known as extravasation. The new tumor then can spread to this secondary location and thrive. The metastatic cycle is thus reliant on the disruption of cell adhesion, which results in the cell's separation from the parent tumor, and then the cell's capacity to achieve a motile phenotype via alterations in cell-matrix interaction [22].

## 4. Performance Analysis

**Figure 2:**
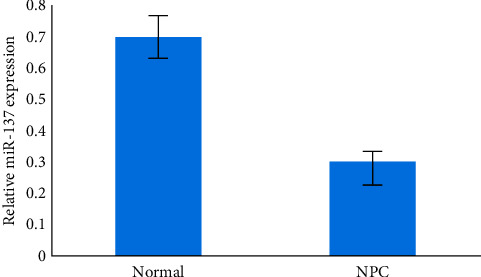
Comparative of normal and NPC.

We used qRT-PCR to demonstrate that miR-137 production was downregulated in NPC materials as compared to normal nasopharyngeal epithelial tissues, *P* < 0.05, as indicated in Figure 2.

**Figure 3:**
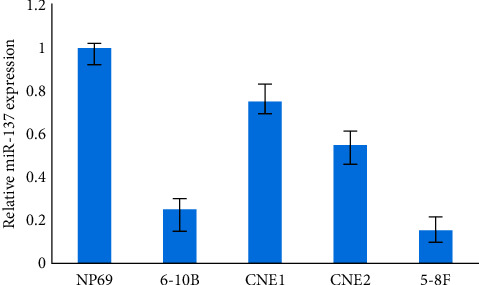
miR-137 is downregulated in NPC.

Moreover, as compared to normal nasopharyngeal epithelial cell line NP69, the production of miR-137 was substantially lower in a set of NPC cell cultures CNE1, CNE2, 5-8F, and 6–10B, *P* < 0.01, as shown in Figure 3. These findings indicate that miR-137 was downregulated in NPC. In vitro, miR-137 suppresses NPC invasion and migration.

**Figure 4:**
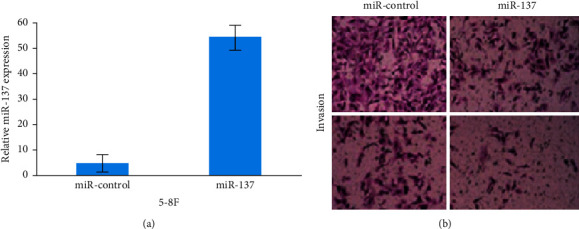
(a) miR-137 vs. 5-8F and (b) cell invasion and migration for miR-control and miR-137.

Figure 4(a) shows that the qRT-PCR for miR-137 was performed on 5-8F cells that had been transfected with the appropriate miRNA vectors.  Figure 4(b) shows that the increased levels of miR-137 in 5-8F cells reduced cell invasion and migration as assessed by transwell experiments.

**Figure 5:**
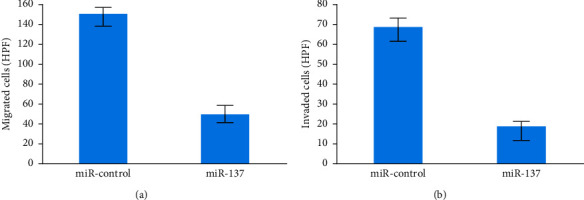
(a) Migrated cells level and (b) invaded cells level.

To demonstrate that miR-137 overexpression substantially reduced the (i) migration of 5-8F and (ii) invasion of 5-8F cells, we used transwell invasion and migration experiments as shown in Figures 5(a) and 5(b).

**Figure 6:**
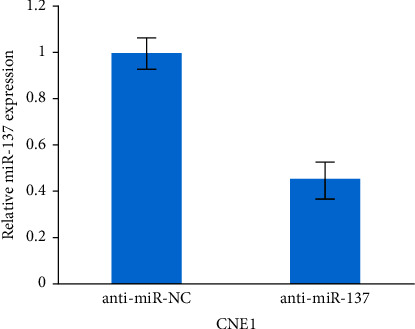
miR-137 versus CNE1.

Figure 6 shows that the qRT-PCR for miR-137 was performed on CNE1 cells that had been treated with inhibitors of miR-137 anti-miR-137 or a control sample.

**Figure 7:**
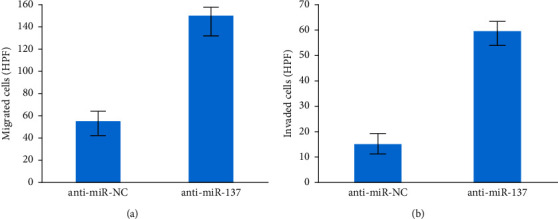
(a) Migrated cells and (b) invaded cells.

To demonstrate that anti-miR-137 overexpression substantially reduced the (i) migration and (ii) invasion cells, we used transwell invasion and migration experiments as shown in Figures 7(a) and 7(b).

## 5. Conclusions

Due to the obvious nature of EMT, it is possible to use it to change effectively. The treatment approaches may be divided into three groups: (i) preventing EMT initiation, (ii) removing cancer cells that are undergoing EMT, and (iii) reversing the EMT in the other direction, that is, MET, for example, since miRNAs play a key role in EMT-induced drug-resistant. Targeted miRNAs in cancer therapy may be a viable strategy. It is argued that MiRNAs may be utilized not only as possible diagnostic or prognostic indicators but also as therapeutic agents. Targeting miRNAs to counteract cancer's aggressive characteristics may have far-reaching therapeutic consequences. Off-target effects and a lack of an appropriate delivery method are still issues with miRNA-based treatments. Because of their rapid excretion, improper intracellular release, low biostability, endosomal escape, poor cellular absorption, and immunogenicity, miRNA administration in vivo remains a problem. As a result, unless these issues are addressed, miRNA-based therapies will not be clinically accessible for cancer therapy.

## Figures and Tables

**Table 1:** Description of genes involved in qRT-PCR.

Name	Forward	Reverse
GAPDH	5′-ATG GTG AAG GTC GGT GTG AAC-3′	5′-TGT AGT TGA GGT CAA TGA AGG-3′
ERR*α*	5′-CCA CTA TGG TGT GGC ATC CTG T-3′	5′-GGT GAT CTC ACA CTC GTT GGA GG-3′
ERR*β*	5′‐GAC ATT GCC TCT GGC TAC CAC T‐3′	5′‐CTC CGT TTG GTG ATC TCG CAC T‐3′
ERR*γ*	5′‐CGC AGG ATA GAT GCG GAG AAC A‐3′	5′‐TTC AGC CAC CAA CAA ATG TGA GAC‐3′

## Data Availability

The data sets used and/or analyzed during the current study are available from the corresponding author on reasonable request.
